# Died of Vaccination

**Published:** 1894-09

**Authors:** 


### "DIED OF VACCINATION."

A piece of white crape is tied on the door of the store at 1508 South Street, Philadelphia, on which is pinned a piece of paper bearing the words, "Jacob H. Wicks, Jr., died June 10th, of vaccination."

Jacob H. Wicks, Jr., was the son of Jacob H. Wicks, a grocer, who lived over the store. His eleven-year-old son went to the James Pollock School, at Fifteenth and Fitzwater Streets. Three weeks ago, when the Board of Health insisted upon the vaccination of all children in the public schools, the child was told that he could not continue at school unless he was vaccinated.

Mrs. Wicks, the boy's mother, refusing the aid of the city physician, called in their family doctor. The child was innoculated with the virus, which quickly took effect.

### SICKNESS AND DEATH FOLLOWED.

Last Wednesday he became very ill and was forced to leave school. Symptoms of cellulitis appeared on Thursday, and the pain became greater, the sore spread, and the limbs began to swell. On Friday the symptoms appeared alarming. Dr. James Graham called in a surgeon for consultation and the case seemed so serious that on Sunday it was decided to lance the swelling. This operation, however, proved unsuccessful, and the boy died at 8.45 P. M. the same evening.

Dr. James Graham, who vaccinated the child, when seen last evening at his house, 1502 Spruce Street, said: "The boy's death was not due directly to vaccination, although it was indirectly the cause of his decease. Cellulitis was the direct cause. If he had not been vaccinated, however, cellulitis would not have developed. It had existed in a mild form for a number of days, but did not assume a defined form until Thursday. It terminated fatally on Sunday. Vaccination causes an irritation of the skin. People have died from a mosquito bite and cellulitis may be caused by a mere scratch. Thousands of children have been vaccinated. If that number had been scratched without being innoculated with virus and some dirt or other foreign substance had been put into the sore accidentally, as with the finger-nails, while the same damp, chilly weather had existed, probably the lives of numbers of them would have been put in jeopardy."

### THE ONLY DEATH IN MANY CASES.

"Do you know of any other deaths due indirectly to vaccination?" the doctor was asked.

"In the twenty-seven years of my practice I have vaccinated a very large number of children and this is the only death.

The virus was obtained from a New England firm which I consider produces the best obtainable. I only had it three or four days and used other virus from the same source and obtained at the same time successfully. I haven't used virus from scabs for years. The sore was lanced Sunday after consultation with one of the best surgeons in Philadelphia."

The doctor refused to give any explanation of the presence of the foreign matter which had caused cellulitis, but intimated that it might have resulted from the child's scratching the sore with dirty finger-nails. He had used every possible precaution himself to provide against accident.—*Philadelphia Inquirer*, Tuesday, June 12th, 1894.

